# A CRISPR way for accelerating cereal crop improvement: Progress and challenges

**DOI:** 10.3389/fgene.2022.866976

**Published:** 2023-01-06

**Authors:** Umer Basu, Syed Riaz Ahmed, Basharat Ahmad Bhat, Zunaira Anwar, Ahmad Ali, Aqsa Ijaz, Addafar Gulzar, Amir Bibi, Anshika Tyagi, Suresh M. Nebapure, Chengeshpur Anjali Goud, Shafat Ahmad Ahanger, Sajad Ali, Muntazir Mushtaq

**Affiliations:** ^1^ Division of Entomology, ICAR-Indian Agricultural Research Institute, New Delhi, India; ^2^ Nuclear Institute for Agriculture and Biology College, Pakistan Institute of Engineering and Applied Sciences (NIAB-C, PIEAS), Faisalabad, Pakistan; ^3^ Department of Bioresources, University of Kashmir, Srinagar, India; ^4^ National Key Laboratory of Crop Genetic Improvement, College of Plant Science and Technology, Huazhong Agricultural University, Wuhan, China; ^5^ Division of Plant Pathology, Faculty of Agriculture, Sher-e-Kashmir University of Agricultural Sciences and Technology of Kashmir, Wadura Sopore, India; ^6^ Department of Plant Breeding and Genetics, Faculty of Agriculture Sciences, University of Agriculture Faisalabad, Faisalabad, Pakistan; ^7^ Department of Biotechnology, Yeungnam University, Gyeongsan, South Korea; ^8^ Institute of Biotechnology, Professor Jayashanker Telangana State Agriculture University, Hyderabad, India; ^9^ ICAR-National Bureau of Plant Genetic Resources, Division of Germplasm Evaluation, Pusa Campus, New Delhi, India

**Keywords:** CRISPR/Cas, cereals, food security, genome editing, crop improvement, TALENs, base editing, prime editing

## Abstract

Humans rely heavily on cereal grains as a key source of nutrients, hence regular improvement of cereal crops is essential for ensuring food security. The current food crisis at the global level is due to the rising population and harsh climatic conditions which prompts scientists to develop smart resilient cereal crops to attain food security. Cereal crop improvement in the past generally depended on imprecise methods like random mutagenesis and conventional genetic recombination which results in high off targeting risks. In this context, we have witnessed the application of targeted mutagenesis using versatile CRISPR-Cas systems for cereal crop improvement in sustainable agriculture. Accelerated crop improvement using molecular breeding methods based on CRISPR-Cas genome editing (GE) is an unprecedented tool for plant biotechnology and agriculture. The last decade has shown the fidelity, accuracy, low levels of off-target effects, and the high efficacy of CRISPR technology to induce targeted mutagenesis for the improvement of cereal crops such as wheat, rice, maize, barley, and millets. Since the genomic databases of these cereal crops are available, several modifications using GE technologies have been performed to attain desirable results. This review provides a brief overview of GE technologies and includes an elaborate account of the mechanisms and applications of CRISPR-Cas editing systems to induce targeted mutagenesis in cereal crops for improving the desired traits. Further, we describe recent developments in CRISPR-Cas–based targeted mutagenesis through base editing and prime editing to develop resilient cereal crop plants, possibly providing new dimensions in the field of cereal crop genome editing.

## Introduction

Natural disasters and climate change have significantly harmed our agricultural systems in recent decades. In the near future, agriculture may face immense challenges in feeding a population, that is, likely to rise to 9 billion by 2050. Therefore, in order to fulfill the requirements of food supply to the global human community, scientific communities have largely focused on modern technological interventions to modify major crops for improved yield and resilient qualities. Cereal crops are considered to be the main energy and protein source for humans because they provide approximately 50% of dietary energy globally, especially in developing countries, where the contribution is higher (Borrill, 2020; Poutanen et al., 2022). Cereals are high in dietary fiber and contain adequate carbohydrates, protein, lipids, fats, vitamins, and minerals. With these nutritional values, health benefits, and production, cereals have been a staple in our diet since the establishment of agriculture farming. Therefore, cereals are vital to global food and nutritional security. Abiotic and biotic stresses are the most devastating factors for cereal crop production, affecting all growth stages and posing serious threat to global food security. In this regard, modern developments in GE technology have accelerated a transition to precision breeding for crop improvement, by making selective and precise genetic alterations in crops. A series of technologies known as GE enable researchers to change any DNA. GE makes it possible to change, add, or remove a particular sequence from the genome of any living organism. Homologous recombination is the basis of genome engineering; however, its occurrence at low frequencies limits the editing efficiency (Chen et al., 2019). To improve the editing frequency, researchers have improved the utility of programmable endonucleases that generate DNA double-stranded breaks (DSB) at target sites. The evolution of various GE technologies such as transcription activator-like effector nucleases (TALEN) and zinc finger nuclease (ZFN) have been previously used in the intended modification of human, animal, and plant cell genomes (Shukla et al., 2009; Zhang et al., 2010; Davies et al., 2017; Adli, 2018; Kannan et al., 2018; Manghwar et al., 2019; Arimura et al., 2020; Yasumoto et al., 2020; Dong and Ronald, 2021). ZFN is a site-specific GE approach which combines the DNA binding domains of zinc-fingers (ZFs) with the restriction endonucleases *Fok*I (Kim et al., 1997). ZF domains that have been custom engineered are important for site-specific mutagenesis (Carroll, 2008; Urnov et al., 2010). ZFs have been developed to target unique DNA sequences at specific loci in order to decrease off-target effects since ZFs have been implicated in target site recognition and binding efficacy for a wide range of DNA sequences. *Fok*Tandem array of Cys_2_-His_2_ zinc fingers (ZFNs) have been generated, with each unit containing −30 amino acids bound to a single atom of zinc; each domain aids in the recognition of, and binding to particular nucleotide triplets in the target sequence. Combining several ZFs to generate an array of DNA-binding ZFs might improve the affinity and selectivity for recognition of target DNA sequences (Choo and Isalan, 2000; Pabo et al., 2001; Segal et al., 2003). Although the use of ZFN for GE is accompanied by numerous limitations, it has been successfully used in cereals such as maize (Shukla et al., 2009) and rice (Cantos et al., 2014). Moreover, Sangamo BioSciences and Sigma Aldrich (licensed ZFN provider) have designed ZFNs with minimal off-targeting, as illustrated by the efficacy and specificity of the ZFNs to several crop plants including corn, canola, and wheat (Davies et al., 2017). Owing to its complexity and off-targeting, ZFN-based gene editing entails optimization of the stability, and targeted mutagenesis devoid of any off-target risks. Interestingly, specific sequence nucleases such as TALENs and Cas9 have been proposed, with a simplistic construct design and superior efficiencies than ZFN.

TALENs are synthetic hybrid proteins comprising a TALE DNA-binding domain linked to a *Fok*I nuclease domain (Zhang et al., 2015). TALEs are proteins containing a DNA-binding domain made up of a string of tandem repeats that are secreted by plant bacterial pathogens of the genus *Xanthomonas* after infection of the host (Mak et al., 2013). Each domain is made up of a sequence of 33–35 repeating amino acids that differ significantly at positions 12 and 13, exhibiting hypervariability. The 13th amino acid is responsible for interactions with a specific DNA base, and the 12th amino acid stabilizes this bonding (Deng et al., 2012). These sites are referred to as RVDs (repeat variable diresidues) (Mak et al., 2013; Wei et al., 2013; Lau et al., 2014). The RVD type and order (in the TALE repeat) determine the target specificity of TALE. If the TALE repeats are interchanged with different RVDs, this results in novel specificities. In 2009, two separate research groups demonstrated that RVDs were accountable for the attachment of certain nucleotides at the TALE target site, in accordance with a simple code (Boch et al., 2009; Moscou and Bogdanove, 2009). Each RVD identifies a 1-bp sequence rather than the 3-bp motif identified by zinc fingers, hence the sequence specificity of TALENs may be designed more accurately than in ZFNs (Son and Park., 2022). The RVDs Asn-Ile (NI), Asn-Asn (NN), His-Asp (HD), and Asn-Gly (NG) recognize the nucleotides A, G/A, C, and T, respectively (Christian et al., 2012). In accordance with the DNA-binding selectivity code, TALEs can be customized to attach to any arbitrary DNA sequence and joined to the endonuclease site of *Fok*I to produce a TALEN. To create site-specific DNA double strand breaks (DSBs), twin TALENs addressing sense and antisense strands are required, as *Fok*I requires the formation of a dimer for DNA cleavage. Site-specific indel mutations can be induced by localized DSBs through the error-prone non-homologous end-joining (NHEJ) repair pathway. By using a sister chromatid or an external homologous DNA template, homologous recombination becomes another method for repairing DSBs that enables highly precise editing such as the insertion or replacement of genes at the target areas. However, due to their reliance on a restricted number of loci, the production of specialized enzymes, the high cost of a particular protein domain assembly, the usage of specific monomers in vector creation, and the accompanying off-target implications, ZFN and TALEN have become obsolete. Moreover, as molecular biology and plant breeding have changed dramatically, new CRISPR tools (CRISPR/Cpf1, prime editing, and base editing) have been created to modify the genomes of plants accurately, effectively, and swiftly (Tan et al., 2019; Haroon et al., 2022).

The CRISPR/Cas9 system leverages RNA-guided DNA cleavage to execute genome editing and is extremely efficient compared with prior genome editing systems such as ZFNs and TALENs, which rely on protein-guided sequence-specific DNA recognition and cleavage (He and Zhao, 2020; Li et al., 2020; Mushtaq et al., 2021b). Prior genome editing CRISPR-Cas reagents such as sgRNA, Cas proteins, and DNA must be delivered to the plants. Transfection of protoplasts, biolistic transformations, or *Agrobacterium*-mediated processes are used as delivery systems. CRISPR systems may be categorized into two classes, each of which possesses six types and 19 sub-types (Shmakov et al., 2017). Class 2 systems have become mainstream in genome editing technology because they require a single Cas protein, whereas Class 1 systems employ a multi-subunit Cas complex. Hence, the most explored and utilized method is a Class 2, type II CRISPR/Cas9 system, which uses a single Cas protein from *Streptococcus pyogenes* (SpCas9). Cas9 is an endonuclease that was identified in *S. pyogenes* which possesses RuvC and HNH nuclease domains. Its cleavage specificity is determined by CRISPR RNA (crRNA), formed from a CRISPR array that encapsulates short segments of foreign DNA molecules encountered by the bacteria. This CRISPR system is then transformed into tiny crRNAs that drive Cas9 to the target sequence (such as foreign DNA), resulting in Cas9-directed cleavage of both non-target and target DNA strands inside the crRNA-target DNA complex. In this mechanism, trans-activating crRNA (tracrRNA), which acts as a connection between crRNA and Cas9, is required for maturation of the crRNA. The CRISPR/Cas9 system in *S. pyogenes* has been curtailed to just two components: Cas9 and a small RNA. A single-stranded, single-guide RNA (sgRNA) emulates the crRNA:tracrRNA duplex, and exhibits a unique 20-bp sequence before the adjacent protospacer motif (PAM) with the sequence NGG, which is required for Cas9 compatibility (Zhang et al., 2017). The sgRNA and Cas9 complex attaches to a specific target site present on genomic DNA, permitting the complex to cleave the complementary site, resulting in a double-stranded DNA break (DSB) (Son and Park., 2022). Following the creation of a DSB, two main paths exist: non-homologous end-joining (NHEJ) and homologous recombination (HR) (Moore and Habber, 1996; Song et al., 2021). Since NHEJ-mediated knockouts provide a very precise and efficient method of suppressing genes of interest, the CRISPR/Cas9 system is ideally attuned for plant breeding. Homology directed repair (HDR) can be employed in scientific research and agriculture for gene substitution, protein tagging, and gene stacking (Nouspikel, 2009; Malzahn et al., 2017).

The current review provides an in-depth understanding of GE technologies and their role in cereal crop improvement. A very deep insight into the applicability, precision, and efficiency of CRISPR/Cas GE techniques is offered. Moreover, we have also profoundly discussed recent advances in genome engineering through an understanding of base editing and prime editing as forefront technologies for crop improvement.

## Technical prelude to evolution of GE technologies

Meganucleases, sometimes called homing endonucleases, are restriction enzymes almost always found in all microorganisms. Meganucleases were the first class of endonucleases used from 1970 to 1980 to produce site-specific double-strand DNA breaks (Jacquier and Dujon, 1985). These hybrid restriction enzymes, which bind the cleavage domain *Fok*I to a customized zinc-finger protein (ZFP), have been utilized to introduce a range of unique changes to eukaryotic cell genomes. They are known to recognize and cleave specific DNA sequences (18–30 bp) to produce double-strand breaks. The resultant double-strand DNA breaks lead to a wide range of DNA modifications such as point mutation, deletion, or insertions (Daboussi et al., 2015). This class of endonucleases is not highly efficient in recognizing site-specific sequences. A challenge for engineering meganucleases is the overlap of cleavage and DNA binding domains. If the sequence of an amino acid is altered in order to gain novel DNA sequence specificity, the catalytic activity of the enzyme is often compromised. However, in recent years, scientists have made tremendous efforts in engineering a variety of meganucleases to cleave specific DNA targets. Nowadays, a number of the engineered meganucleases are used to create genomic modifications in crops for agronomically important traits (Daboussi et al., 2015). ZFNs are hybrid endonucleases and powerful GE tools to introduce double-strand breaks (DSBs) in target genomes, which is usually followed by error-prone non-homologous end joining (NHEJ) repair to create insertions or deletions at the cleavage site. The first report of GE by ZFN in plants was described by knocking-in a herbicide tolerance gene *via* disruption at the *Inositol Phosphokinase1* (*IPK1*) locus to purposefully reduce inorganic phosphate levels in growing seeds as part of an effort to minimize phytate levels in plants (Shukla et al., 2009). The *SSIVa* locus was altered in transgenic rice, impacting grain fullness, starch content, and plant height (Jung, et al., 2018). Several reports demonstrate the successful application of ZFN to modify, add, and disrupt plant genes (Durai et al., 2005; Papworth et al., 2006). Imidazolinone herbicide resistance was accomplished by GE, based on the use of ZFNs in allohexaploid wheat to target the *acetohydroxyacid synthase* (*AHAS*) encoding gene (Ran et al., 2018).

For precise genome editing, TALENs have been used instead of ZFNs due to their simple assembly, high success rate, availability of powerful resources, and decreased off-targeting. The discoveries of the Transcription Activator-Like Effector (TALE-DNA binding domains) and TALENs (TALE nucleases) were important breakthroughs in the field of genetic engineering. TALENs have allowed scientists to create double stand DNA breaks, introducing DNA modifications, gene knockout, and gene knock-in. The speed and ease of creating TALEN reagents has made it possible for a large number of labs to make target-specific alterations in genes of interest, cells, or organisms using the available transformation methods (Cermak et al., 2015). TALEs (TAL effectors) can be virulence factors, plant-recognized avirulence factors, or both (Bogdanove et al., 2010). These proteins imitate transcription factors once they attach to the DNA sequence and can control the activation of target gene(s) (Becker and Boch. 2021; Saurabh, 2021). Researchers decided to use it as a tool for gene editing by creating two hybrid TALE nucleases, each containing a DBD and the catalytic domain *Fok*I. This hybrid chimeric nuclease attaches to DNA and produces double-strand DNA breaks (DSBs). The majority of these DSBs are fixed by the NHEJ mechanism with insertions or deletions (indels), leading to an altered genome. The use of TALENs for genome editing was shown to be effective in cereal plants, including maize (Yu et al., 2014; Kelliher et al., 2017), rice (Shan et al., 2015), wheat (Zong et al., 2017; Luo et al., 2019), barley (Gurushidze et al., 2014), and other cereal crops. Applications of ZFNs and TALENs in phenotypic and nutritional enhancement are summarized in tabulated form (Table 1). However, there are some limitations associated with TALENs such as problems in editing a methylated target site, successful transmission with a vector, off-target effects, non-specific binding ability, and large size, necessitating further development of this technology (Pennisi, 2013; Mahfouz et al., 2014; Mushtaq et al., 2018; Razzaq et al., 2019; Ansari et al., 2020).

**TABLE 1:** ZFNs and TALENs for nutritional and phenotype improvement in cereals.

Crop	Gene editor	Gene targeted	Improvement	Method	References
Rice (*Oryza sativa*)	ZFNs	*OsQQR*	Trait stacking	HDR	Cantos et al. (2014)
	TALEN	*OsDEP1*, *OsBADH2, OsCKX2*, and *OsSD1*	Gene knockout	NHEJ	Shan et al. (2013)
	TALEN	*OsBADH2*	Fragrant rice	NHEJ	Shan et al. (2015)
	1.1.1 TALEN	*OsMST7* and *OsMST8*	Gene knockout	NHEJ	Zhang et al. (2016)
	TALEN	*Os11N3* (*OsSWEET14*)	Disease resistance	NHEJ	Li et al. (2012)
Maize (*Zea mays*)	ZFNs	*ZmIPK1*	Herbicide tolerant and phytate-reduced maize	HDR	Shukla et al. (2009)
	ZFNs	*ZmTLP*	Trait stacking	HDR	Ainley et al. (2013)
	TALEN	*ZmMTL*	Induction of haploid plants	NHEJ	Kelliher et al. (2017)
	TALEN	*ZmIPK1A*, *ZmIPK*, and *ZmMRP4*	Phytic acid synthesis	NHEJ	Liang et al. (2014)
	TALEN	*ZmGL2*	Reduced epicuticular wax in leaves	NHEJ	Char et al. (2015)
Barley (*Hordeum vulgare*)	TALEN	*HvPAPhy-a *	Phytase activity	NHEJ	Wendt et al. (2013)
	TALEN	*BAR*	Bialaphos resistance	__	Gurushidze et al. (2014)

The flexibility of ZF and TALE DNA binding domains allows them to assemble or reprogram in a specific fashion and to recognize a particular site in the targeted genome, provided a significant advantage to ZFN and TALEN tools for genetic engineering, compared with the CRISPR-Cas9 GE system (Musunuru, 2017). In plant GE for agricultural enhancement, these two techniques have been widely employed (Forsyth et al., 2016; Ran et al., 2018; Shan et al., 2018). However, due to off-target occurrences, tedious build designs, poor efficiency, and expensive cost, their applicability to plant GE has been confined (Cermak et al., 2011; Puchta, 2017; Khan., 2019). Because of these constraints, a new, low cost, precise, and specific technology called CRISPR/Cas9 was developed as a flexible tool for biological studies to understand gene functions and crop enhancement (He et al., 2018; Lee et al., 2018; Mushtaq et al., 2018; Soyars et al., 2018; Manghwar et al., 2019; Mushtaq et al., 2019; Selma et al., 2019; Mushtaq et al., 2020; Mushtaq et al., 2021a; Mushtaq et al., 2021b; Mushtaq and Molla, 2021).

## CRISPR for accelerated cereal crop improvement

**FIGURE 1:**
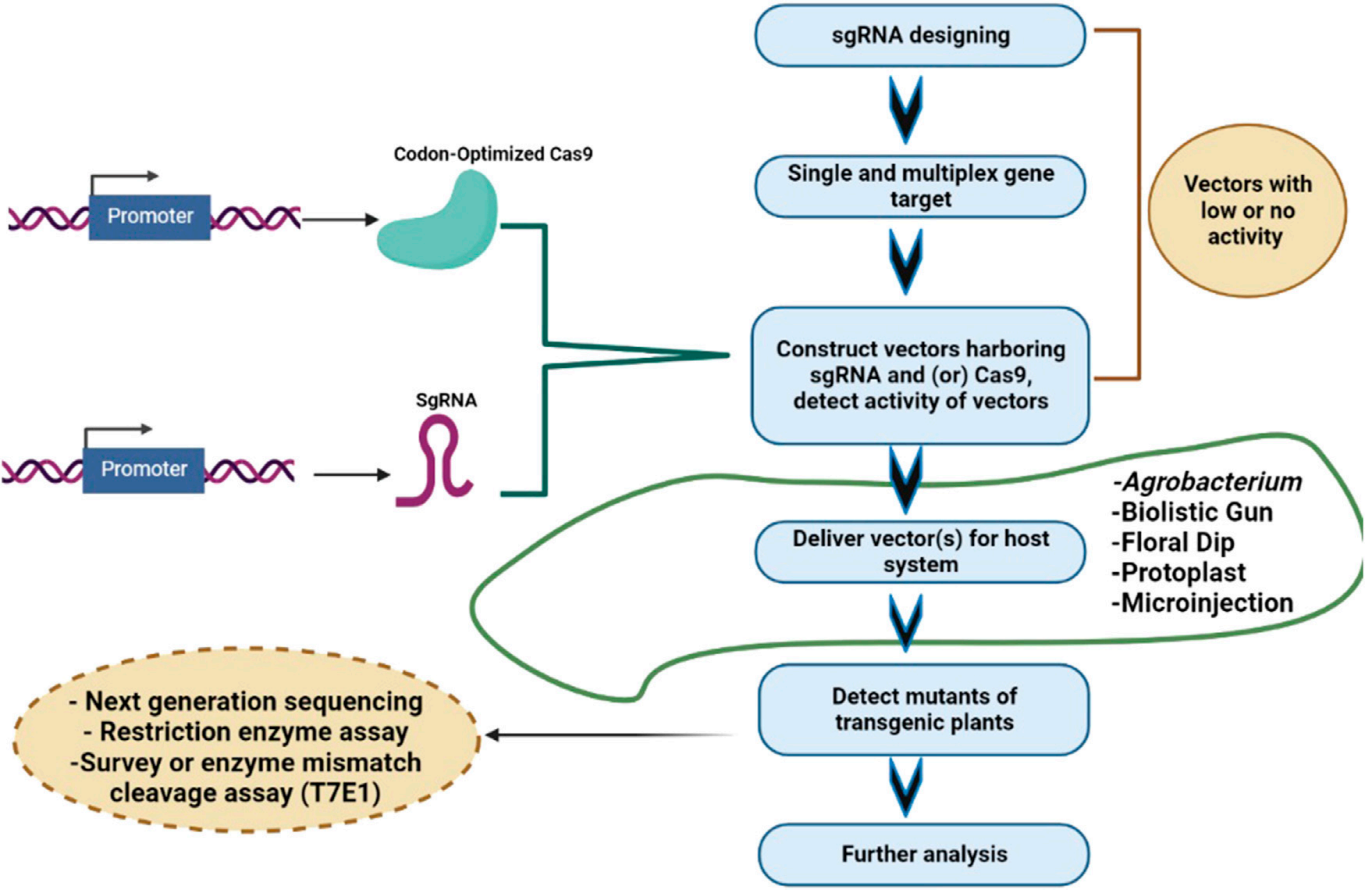
Steps involved in CRISPR/Cas9-based gene editing of cereals. Selection of target gene and gRNA design; cloning of Cas9 and gRNA in a suitable vector; vector delivery into the plants *via Agrobacterium tumefaciens* or particle bombardment, and screening of mutant/edited cereal crops using Sanger sequencing/NGS/RE/PCR.

**FIGURE 2:**
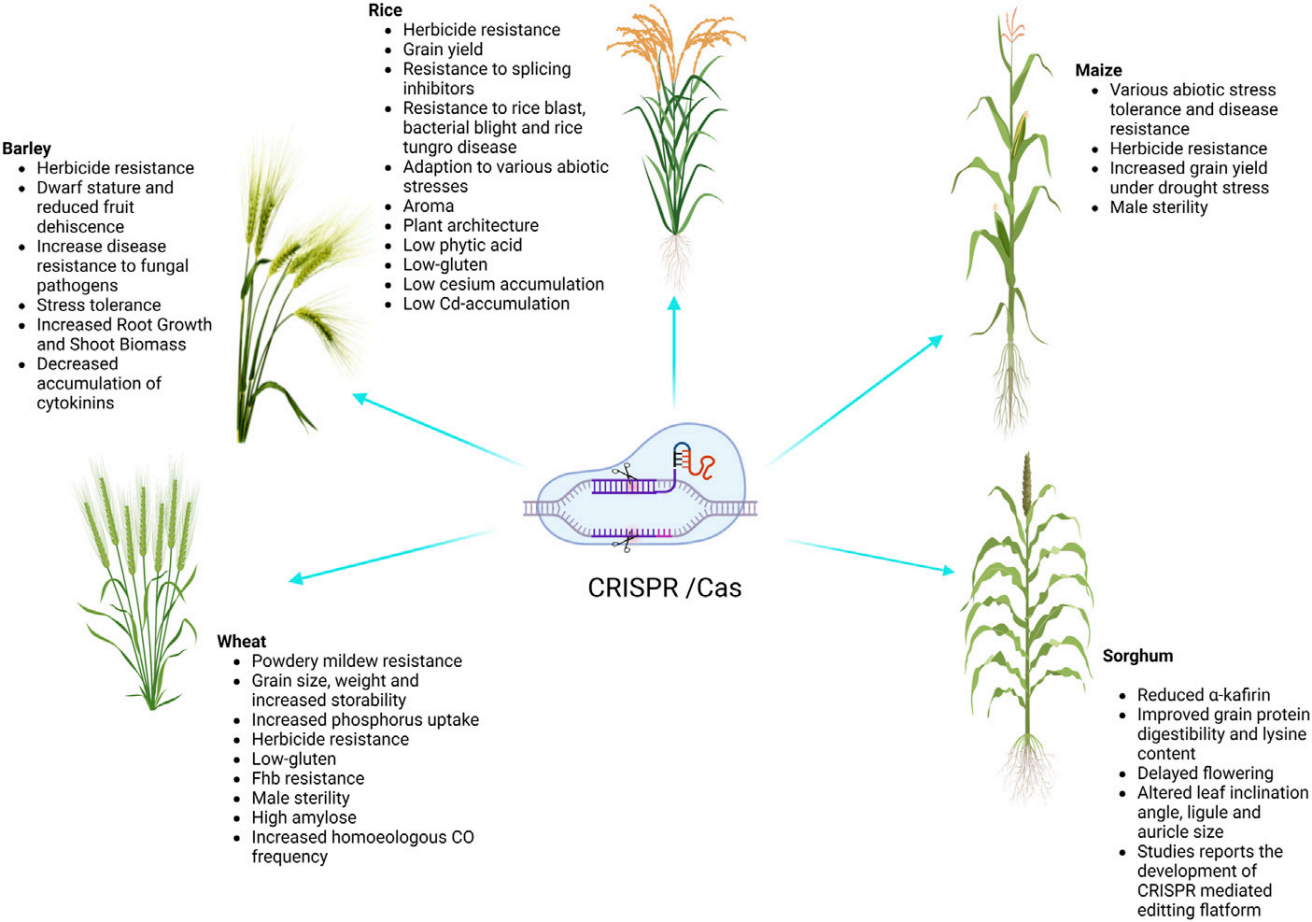
Successful application of CRISPR/Cas genome editing in cereals.

Cereal crops are treated as a predominant food and a source of energy due to their supply of essential nutrients in the human diet. It has been estimated that more than 90% of global food production is derived from cereal crops. Rice and wheat are the staple foods of India, Bangladesh, Pakistan, and Afghanistan. CRISPR/Cas9 technology is the prime choice to address the growing demand for cereal crops, owing to its high accuracy and efficiency. CRISPR/Cas9 technology is capable of enhancing tolerance against biotic and abiotic stresses in cereal crops. A schematic workflow of CRISPR/Cas9-based GE in cereals is shown in Figure 1. Details of CRISPR/Cas9 technology applications in cereal crops are categorized in detail in Table 2. Cereal crops in which CRISPR/Cas-based GE has been used to modify different traits are also proposed (Figure 2). For clarity, and in-depth use of this versatile technology, we have considered the viability of CRISPR/Cas9 system-based GE in each individual cereal crop in the following sections.

**TABLE 2:** Applications of CRISPR/Cas9 system in cereal crops.

Crop	Type of study	Targeted gene	Method	sgRNA promoter	Cas9 promoter	Editing efficiency (%)	References
Wheat (*Triticum aestivum*)	Genome editing	*Tainox* and *Tapds*	*Agrobacterium-*mediated	CaMV 35S	CaMV 35S	11–12	Upadhyay et al. (2013)
	Genome editing	*TaGLW7*, *TaGW8*, *TaGW2*, and *TaCKX2-1*	*Agrobacterium-*mediated	TaU6.1, TaU6.2, TaU6.3, and TaU6.5	ZmUbi	64.3	Zhang et al. (2019a)
	Genome editing	*TaGASR7A1*, *TaGASR7 B1*, and *TaGASR7D1*	Biolistic	TaU6	CaMV 35S	5.2	Hamada et al. (2018)
	Knockout	*TdGASR7*	Biolistic	TaU6	2×CaMV 35S	1.8	Liang et al. (2018)
	Knockout	*TaGW2B1*, *TaGW2D1*, and *TaGW2A1*	Protoplast transfection using RNP	__	__	0–4.4	Liang et al. (2017)
	Gene editing	*TaABCC6*, *TaNFXL1*, and *TansLTP*	Protoplast transformation	TaU6	CaMV 35S	NA	Cui et al. (2019)
	knockout	*GW2-B*, *PinB-D*, and *ASN2-A*	Protoplast transformation using RNP	__	__	0–36	Brandt et al. (2020)
	Genome editing through transient expression	*TaGASR7A1*, *TaGASR7B1*, and *TaGASR7D1*	Particle bombardment	TaU6	Ubi	1.1–5	Zhang et al. (2016)
	Site-directed mutagenesis	*TaMLOD1*, *TaMLOA1*, and *TaMLOB1*	Particle bombardment	U6	Ubi1	3.4–6	Wang et al. (2014)
	Site-directed mutagenesis	*LOX2*	Protoplast transformation	TaU6	2×CaMV 35S	∼1	Shan et al. (2014)
	Functional genomics	*TaPDS*	*Agrobacterium-* mediated	TaU6	ZmUbi	11–17	Howells et al. (2018)
Sorghum (*Sorghum bicolor*)	Gene editing	*TaLox2* and *TaUbiL1*	Electroporation	TaU6	Ubi1	2.2	Bhowmik et al. (2018)
	Gene editing	*CAD* and *PDS*	Particle bombardment	U3	ZmUbi	NA	Liu et al. (2020)
	Gene editing	*K1c*	*Agrobacterium-*mediated	TaU3	ZmUbi	14.1–78.3	Li et al. (2018)
	Knockout	*SbFT* and *SbGA2ox5*	*Agrobacterium-*mediated	U6P.1 and U6P.2	ZmUbi	33.3–83.3	Char and Yang. (2019)
	Targeted mutagenesis	*StALS1*	*Agrobacterium-*mediated	U6	2×CaMV 35S	5–60	Butler et al. (2015)
	Knockout	*Sb-CENH3*	*Agrobacterium-*mediated	U6	ZmUbi	37–40	Che et al. (2018)
	Functional genomics	*DsRED2*	*Agrobacterium-*mediated	U6	OsActin1	NA	Jiang et al. (2013)
Rice (*Oryza sativa*)	Genome editing	*TaLOX2*	*Agrobacterium-*mediated	OsU3	2×CaMV 35S	∼1	Shan et al. (2014)
	Gene editing	*OsPMS3*, *OsYSAOsDERF1*, *OsMYB1*, *OsMSH1*, *OsPDS*, *OsSPP*, *OsEPSPS*, *OsMYB5*, and *OsROC5*	*Agrobacterium-*mediated	U3 and U6	ZmUbi. CaMV 35S	>35	Ma et al. (2015)
	Gene editing	*SBEI* and *SBEIIb*	*Agrobacterium-*mediated	OsU3	Ubi1	26.7–40	Sun et al. (2017)
	Knockout	*elF4G*	*Agrobacterium-*mediated	TaU6	ZmUbi1	30–64	Macovei et al. (2018)
	Knockout	*OsNramp5*	*Agrobacterium-*mediated	OsU6 and OsU3	Ubi1	13.6–35	Tang et al. (2017)
	Loss of function	*OsMORE1* and *OsMORE1a*	*Agrobacterium-*mediated	OsU3	ZmUbi	NA	Kim et al. (2022)
	Knockout	*SAPK1* and *SAPK2*	*Agrobacterium-*mediated	OsU3 and OsU6a	CaMV 35S	NA	Lou et al. (2018)
	Gene editing	*OsPIN5b*, *gs3*, and *OsMYB30*	*Agrobacterium-*mediated	U6	CaMV 35S	42–66	Zeng et al. (2020)
	Deletion	*Waxya* and *Waxyb*	*Agrobacterium-*mediated	OsU3 or OsU6	CaMV 35S	8.6–11.85	Liu et al. (2022)
	Gene editing	*RLK*	*Agrobaterium-*mediated	U3	Ubi1	NA	Chen et al. (2022)
	Knockout	*ISA1*	*Agrobacterium-*mediated	U6	CaMV 35S	NA	Shufen et al. (2019)
	Knockout	*Waxy*	*Agrobacterium-*mediated	U6	CaMV 35S	82.7–86.9	Zhang et al. (2018)
	Knockout	*Waxy*	*Agrobacterium-*mediated	U3 and U6a	CaMV 35S	NA	Yunyan et al. (2019)
	Knockout	*OsRR22*	*Agrobacterium-*mediated	OsU6a	UbiH	64.3	Zhang et al. (2019a)
	Knockout	*OsCCD7*	*Agrobacterium-*mediated	OsU3	OsUbi	22.2–64.3	Butt et al. (2018)
	Knockout	*EPSPS*	Protoplast transformation	OsU3 and TaU3	__	2.0–2.2	Li et al. (2016)
	Site-directed mutagenesis	*OsROC5*, Os*SPP*, and Os*YSA*	*Agrobacterium-*mediated	OsU6-2	CaMV 35S	61.1–67.7	Zhang et al. (2014)
	Site-directed mutagenesis	*OsMPK5*	*Agrobacterium-*mediated	U3 and U6	CaMV 35S	3–8	Xie and Yang. (2013)
	Site-directed mutagenesis	*OsPDS*, *TaLOX2*, *OsBADH*, and *OsMPK2*	*Agrobacterium-*mediated	OsU3	CaMV 35S	∼1	Shan et al. (2014)
	Site-directed mutagenesis	*OsMYB1*	Protoplast transformation	OsU3	CaMV 35S	NA	Miao et al. (2013)
	Multiplex editing capability with endogenous tRNA	*OsMPKs*	*Agrobacterium-*mediated	OsU3	OsUbi	6–100	Xie et al. (2015)
	Multiplex GE in dicot andmonocot plants	46 genomic targets	*Agrobacterium-*mediated	OsU3, OsU6 and OsU6c	OsUbi and CaMV 35S	24.7–90	Ma et al. (2015)
Barley (*Hordeum vulgare*)	Functional studies	*WDV1*, *WDV2*, *WDV3*, and *WDV4*	*Agrobacterium-*mediated	WDV	CaMV 35S and ZmUbi	NA	Kis et al. (2019)
	Fragment Deletions and Small Indels	*ENGase*	*Agrobacterium-*mediated	OsU6	ZmUbi	78	Kapusi et al. (2017)
	Gene editing	*NbPDS1*	*Agrobacterium-*mediated	U6	CaMV 35S	NA	Raitskin et al. (2019)
	Gene editing	*Hpt*	*Agrobacterium-* mediated	U6	ZmUbi	20–70	Watanabe et al. (2016)
	Knockout	*HptII*	*Agrobacterium-*mediated	U6	ZmUbi	NA	Lawrenson and Harwood. (2019)
	*Knockout*	*HvMORC1* and *HvMORC6a*	*Agrobacterium-*mediated	HvU3	CaMV 35S	8–81	Galli et al. (2022)
	Knockout	*HvCKX1*	*Agrobacterium-*mediated	HvU3	ZmUbi1	NA	Holubova et al. (2018)
	Knockout	*HvMORC1*	*Agrobacterium-*mediated	HvU3	ZmUbi	38–77.7	Kumar et al. (2018)
	Knockout	*HvCKX1*, *HvCKX3*, and *Nud*	*Agrobacterium-*mediated	TaU6	ZmUbi	18–68	Gasparis et al. (2018)
	Knockout	*GPhsp70*, *GPhsp26*, *GPhsp16.9*, *GPgst*, *GPcrt*, *GPipi*, and *GPpdi*	*Agrobacterium-*mediated	TaU6	ZmUbi	NA	Panting et al. (2021)
Maize (*Zea mays*)	Knockout	*waxy*	Biolistic	TaU6	ZmUbi	1–55.4	Gao et al. (2020)
	Gene editing	*ZmIPK*	*Agrobacterium-*mediated	ZmU3	CaMV 35S	13.1	Liang et al. (2014)
	Gene editing	*ZmHKT1*	*Agrobacterium-*mediated	AtU6-26, OsU3 or TaU3	CaMV 35S or Ubi1	NA	Xing et al. (2014)
	Gene editing	*LIG1*, *MS26*, and *MS45*	*Agrobacterium-*mediated	ZmU6	ZmUbi	6–86	Svitashev et al. (2015)
	Gene editing	*Zmzb7*	*Agrobacterium-*mediated	ZmU3	CaMV 35S	19–31	Feng et al. (2016)
	Gene editing	*PSY1*	*Agrobacterium-*mediated	ZmU6	ZmUbi	10.67	Zhu et al. (2016)
	Gene editing	*CLE*	*Agrobacterium-*mediated	__	__	NA	Liu et al. (2021)

### Wheat

Wheat (*Triticum aestivum* L.) is one of the three main crops grown for human consumption, hence, wheat improvement initiatives for higher yields and improved resilience are crucial for ensuring global food security. Mildew-tolerant wheat varieties have been successfully developed *via* CRISPR/Cas9 to knockout the *TaMLO* gene coding for mildew resistance (Tripathi et al., 2020). This knockout approach resulted in up to 28.50% mutation frequency of the mildew-resistance locus and the crop successfully developed a tolerance (Shan et al., 2014). This experiment initiated interest in the CRISPR/Cas9 system among scientists worldwide to improve cereal crops. Another gene, *TaEDR1*, was known for developing tolerance against powdery mildew well before the advent of CRISPR/Cas9 technology; however, the expression level of this gene required enhancement to achieve improved results, and was later performed using CRISPR/Cas9 (Zhang et al., 2017). Char and Yang (2020) also successfully knocked-out the *TaEDR1* gene *via* CRISPR/Cas9 in 2020 to develop powdery mildew-tolerant cultivars. In addition to this, the CRISPR/Cas9 system was implemented in wheat to induce mutations in the *Tapx1* and *TaLox2* genes, with mutation rates of approximately 9 and 45% achieved, respectively (Shan et al., 2014). *TaDEP1*, *TaNAC2*, *TaGW2*, and *TaGASR7* genes in wheat were knocked-out *via* CRISPR/Cas9 to increase the grain length, grain width, grain area, and grain weight, compared to wild plants (Wang et al., 2018). We have summarized the application of CRISPR/Cas9 technology for targeting numerous genes in wheat to improve various traits (Table 2).

### Rice

Owing to its small genome size, transformability, accessibility to genetic resources, and sequence data, rice (*Oryzae* spp.) was among the first crops to be extensively modified and studied (Biswas et al., 2020). Additionally, genome-wide association studies (GWAS), comparative genomics, and OMICS-based methods have been used to investigate a variety of genes and SNPs linked to agronomically desirable traits. This allows modification of target genes with greater efficiency. Numerous genome engineering experiments have been carried out, and more recently, the rice genome has been edited using CRISPR/Cas9 technology. The CRISPR/Cas9 technique was used to successfully modify the *OsPDS* (*phytoene desaturase*) gene in rice (Banakar et al., 2020). Two sgRNAs (namely *SP2* and *SP1*) were designed to disrupt the *OsPDS* gene, and mutation frequencies of approximately 9% in transgenic and 15% in protoplasts were observed (Shan et al., 2013). Similarly, the mitogen-activated *protein kinase5* (*OsMPK5*) gene in rice was knocked-out using CRISPR/Cas9 to enhance disease resistance, and a mutation frequency of 3–8% was observed (Ma et al., 2015). The CRISPR/Cas9 technology for multiplex GE in rice has been the subject of several investigations (Liu et al., 2020; Xu et al., 2020). An experiment was conducted where multiple sgRNAs were engineered to be expressed under U3 and U6 promoters. The results revealed that multiple GE using CRISPR/Cas9 technology is highly applicable in rice crops (Ma et al., 2015). A group of scientists conducted two experiments using CRISPR/Cas9 on different genes, with different approaches. In the first experiment, two sgRNAs were designed to target the *NAL1* (*narrow leaf*) gene; the results revealed a low mutation rate for this particular gene (Hu et al., 2016). The second experiment was conducted on *ACT1* (*ACTIN1*) and *UQ1* (*UBIQUITIN1*) genes using CRISPR/Cas9. The mutation frequency of both genes was high enough to develop disease-tolerant genotypes (Hu et al., 2018). Due to the outstanding potential of CRISPR/Cas9 to permanently maintain hybrid vigor, plant biologists and the seed industry have shown a strong interest in apomixis (Wang, 2020). Although, the introduction of apomixis traits from wild relatives into major crops has remained ineffective, artificial apomixis has been used as an alternative to fix the hybrid vigor in rice (Biswas et al., 2020). The development of the *MiMe* (*mitosis* instead of *meiosis*) line in rice, which transforms meiosis into mitosis and results in the development of clonal gametes, has produced rice plants that generate functioning diploid gametes with the same genetic makeup as their parent. Apomixis-like clonal seeds are generated when the *MiMe* line in rice is combined with special genome elimination lines, which contain an altered, centromere-specific histone 3 (CENH3). Furthermore, the generation of haploid plants from egg cells can be achieved by either the egg cell–specific expression of *BABY BOOM1* (*BBM1*), or the disruption of *MATRILINEAL* (*MTL*) using CRISPR/Cas9 gene editing technology. Synthetic apomixis is established, and clonal seeds are produced by simultaneously engineering *MiMe* rice lines with altering *BBM1* expression or *MTL* disruption (Kumar et al., 2020). Additionally, multiple research teams have tried to mutate the genes related to cadmium (*OsNramp5*), drought (*OsSAPK2*), and salt (*OsRR22*) stresses, and the resulting altered lines exhibited improved resistances to the respective conditions (Tang et al., 2017; Liu et al., 2018; Liu et al., 2019). Many other studies have also focused on rice and CRISPR/Cas9-mediated genome editing (Table 2). Studies such as these prove that CRISPR/Cas9 can be successfully exploited for improving the tolerance of rice to stresses like salinity.

### Maize

Maize (*Zea mays* L.) is the third most significant crop after rice and wheat, and is one of the most important cereals that can be cultivated in a wide variety of environmental circumstances (Liang et al., 2014). The first report of GE involved targeted disruption of the *IPK1* (*Inositol Phosphokinase1*) locus *via* knock-in of a herbicide tolerance gene using ZFNs (Shukla et al., 2009). On the other hand, the first use of TALENs in maize was a proof-of concept study to generate stable and heritable mutations at the *GL2* (*GLOSSY2*) locus (Char et al., 2020). Furthermore, GE in maize increased significantly with the advent of the CRISPR/Cas9 technology, and the initial investigations were ground-breaking since they were the first to demonstrate multiplex editing as well as DNA-free editing using Cas9/gRNA ribonucleoproteins (RNPs). In the first studies, five loci (*LG1*, *ALS1*, *ALS2*, *MS26*, and *MS45*) were targeted in maize embryos using DNA constructs, delivered by particle bombardment. Mutations were observed at all five target sites (upstream of *LG1*, in the acetolactate synthase genes *ALS1* and *ALS2*, and in the male fertility genes *MS45* and *MS26*), including multiplex mutations in *LG1*, *MS45*, and *MS26*. CRISPR/Cas9 was successfully used to knock out the *ZmIPK* gene in maize, which controls the formation of phytic acid, while two sgRNAs were utilized underneath the expression promoter U6 to knock out the *phytoene synthase* (*PSY1*) gene with a mutation frequency of 10.67% (Zhu et al., 2016). The authors also sequenced the mutated gene to confirm the effectiveness of the mutation. CRISPR/Cas9 was employed in T_0_ maize lines to target the *albino marker* (*Zmzb7*) gene with a mutation frequency of 31% observed (Feng et al., 2016). By targeting the thermosensitive *male-sterile 5* (*ZmTMS5*) gene with three sgRNAs rather than one or two, researchers were able to perform protoplast alterations (Chen et al., 2018). The modified plants presented bi-allelic modification, demonstrating the potential of the CRISPR/Cas9 technology for intended mutagenesis in maize to improve particular traits (Char et al., 2020). Another major success of gene editing is the development of a maize variety with a higher grain yield under drought-prone environments, by employing precise insertion of a *GOS2* promoter inside the 5′-UTR of *ARGOS8* (Shi et al., 2017). These studies demonstrate the comprehensive applications of CRISPR/Cas9 systems for breeding approaches in maize.

### Barley

In terms of global production, barley ranks as the fourth-most significant cereal crop. Due to its diploid genome structure, barley is used as a model plant for *Triticeae* crop species. Barley gene editing has proven to be a reliable, accurate, and affordable approach for quick plant breeding. Early attempts to establish GE in barley used TALENs and did not target a coding area, instead choosing to focus on the promoter region of the phytase *HvPAPhy*-*a* (Wendt et al., 2013). It was suggested that barley was receptive to GE, without producing a large number of primary transformants because, on average, one out of every four plants bearing the selection marker displayed editing activity. In some cases, editing efficiencies were even up to 88%; editing events were screened by methods other than sequencing, therefore, the reported efficiencies may be conservative estimates (Gasparis et al., 2018). Succeeding GE investigations, where targeted DSBs were mostly induced through *Agrobacterium*-mediated use of conventional Cas9, verified that the editing efficiency is not a constraint. Lawrenson et al. (2015) multicopy genes in barley (*Hordeum vulgare*) and *B. oleracea* to investigate the gRNA Cas9 editing method and target specificity requirements. The researchers targeted two copies of the *HvPM19* gene in *H. vulgare* and *B. oleracea* and found Cas9-induced mutations in 23% and 10% of lines, respectively; mutated plants were stunted in the first-generation. Stable Cas9-induced mutations were transferred to T_2_ plants irrespective of the T-DNA composition in both *H. vulgare* and *B. oleracea.* Although the presence of at least one mismatch between both the sgRNA and the non-target gene sequences was observed, off-target activity across both species was discovered. A transgene-free *H. vulgare* plant exhibited mutations in both the target and non-target alleles of *HvPM19*. Multiple successful efforts have been made to alter the drought and other stress-related genes, specifically *TaDREB2* and *TaERF3*, in transient processes in protoplasts, indicating that this can be a quick method to identify specific and off-targets in the designed gRNAs of barley and wheat (Kim et al., 2018).

## Technical advances in base editing and prime editing

### Base editing

Genome-wide association studies (GWAS) revealed that single-base substitutions are often the best way to introduce excellent traits in crop plants. Based on this, several effective techniques have been employed to generate precise point mutations in crop plants to achieve desired results (Zhang et al., 2018). Numerous agronomic traits have been found to be influenced by single alterations in the bases of genes. Gene base conversion is unfortunately not possible using CRISPR/Cas9 technology. Due to this, finding a precise and reliable method for editing crop genomes is essential. Base editing is thought to be a substitute and a more effective strategy. In agricultural plants, base editing is utilized, replacing HDR-mediated gene editing in an efficient and systematic manner (Bharat et al., 2019). It can achieve automated nucleotide substitutions without disrupting genes. Base editing typically involves a combination of an inactive catalytic CRISPR-Cas9 domain (Cas9 variant, Cas9 nickase, or dCas9) and cytosine or an adenosine deaminase domain that transforms one nucleotide base into another (Mishra et al., 2022). Variations in the single base may produce excellent variant traits in crops, thereby helping to accelerate development in crop plants. Without destruction of genes, base editing can recover single nucleotides or base substitutions, thus reducing deletions and insertions. It is an efficient technology to design new characteristics in important crops for achieving global food and nutrition security (Eid et al., 2018).

**FIGURE 3:**
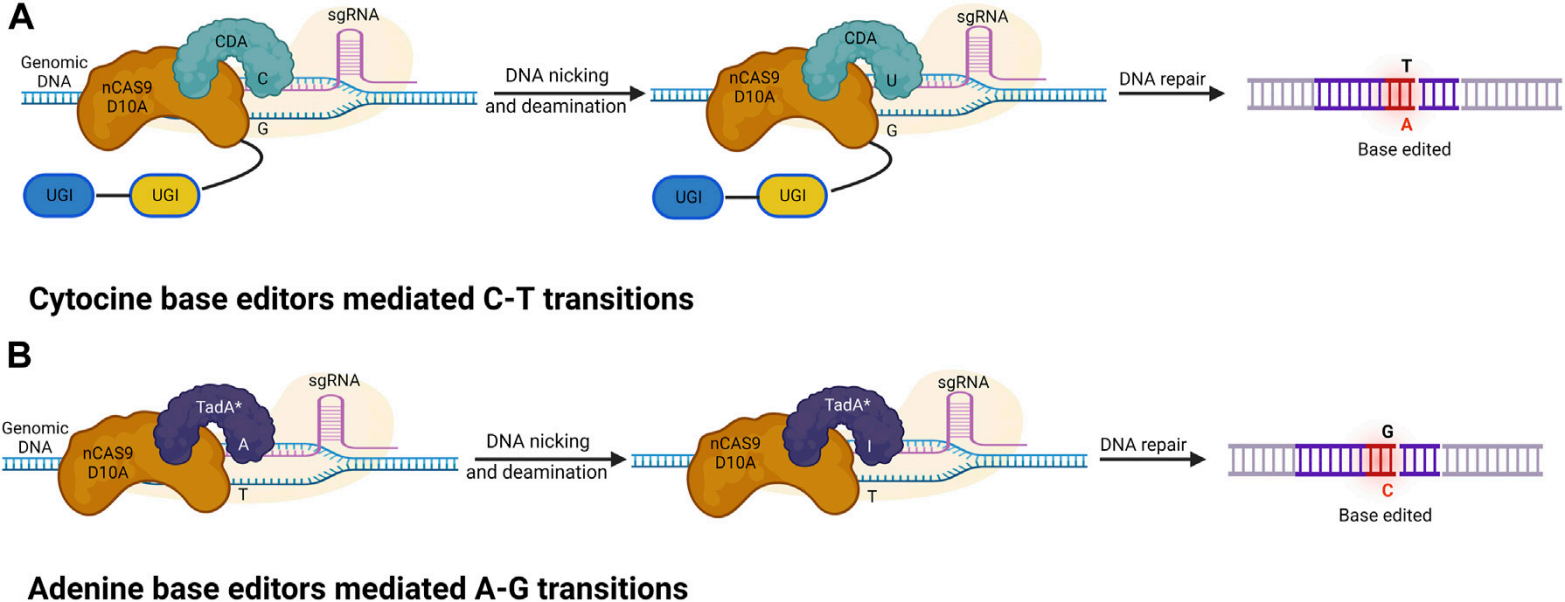
Cytosine and adenine base editors. **(A)** Cytosine base editors (CBEs), composed of a nickase Cas9 (nCas9) fused to a deaminase and UGI; conversion of C-G into T-A base pairs. **(B)** Adenine base editors (ABEs) are composed of a dead (d) or nickase (n) Cas9 (d/nCas9) fused to two TadA, with one evolved to edit adenine in DNA (TadA*) and one wild type (TadA). ABEs convert A-T into G-C base pairs.

A base editor is a chimeric protein composed of a catalytic region and a DNA-targeting module that can deaminate the nucleotide adenine or cytosine in the genome (Komor et al., 2016 and, 2017). In the base editing approach, a combination of the catalytic cytidine deaminase and dCas9 is directed by sgRNA molecules to conduct single-base changes without the formation of double-strand breaks (DSBs) in DNA molecules. The base editor may make single base substitutions, thereby minimizing the frequency of indels. The most commonly used DNA base editors are classified into two types: ABE (Adenine Base Editor) and CBE (Cytosine Base Editor). In recent years these have become effective tools for GE (from C to T and A to G) in eukaryotes (Liu et al., 2016; Qin et al., 2020; Bansal et al., 2021). The base editing method has been effectively improved and verified in various cereal crops including wheat and maize (Table 3). A schematic representation of various Cas9-based base editors are highlighted in Figure 3.

**TABLE 3:** List of genes targeted by cytidine and adenine base editors in cereal crops.

Cereal plant species	Trait improvement	Type of base editor used	Target gene	References(s)
Rice (*Oryza sativa*)	Nitrogen use efficiency	CBE	*NRT1.1B* and *SLR1*	Lu and Zhu (2017)
	Senescence and death	CBE	*OsCDC48*	Zong et al. (2017)
	Nutritional improvement	CBE	*OsPDS* and *OsSBEIIb*	Li et al. (2017)
	Herbicide resistant	CBE	*C287*	Shimatani et al. (2017)
	Pathogen-responsive gene	ABE	*OsMPK6*	Yan et al. (2018)
	Defense response	CBE	*OsRLCK185* and *OsCERK1*	Ren et al. (2018)
	Plant architecture and grain yield	ABE	*OsSPL14*	Hua et al. (2018)
	Herbicide resistance	ABE	*OsACC-T1*	Li et al. (2018)
	Della protein for plant height	ABE	*SLR1*	Hua et al. (2018)
	Herbicide resistance	CBE	*OsSPL14*	Tian et al. (2018)
	Blast resistance	CBE	*Pi-d2*	Ren et al. (2018)
	Herbicide resistance	CBE	*ALS*	Veillet et al. (2019)
	Grain size and yield	ABE	*GL2*/*OsGRF4* and *OsGRF3*	Hao et al. (2019)
	Rice amylose synthesis	ABE	*Wx*	Hao et al. (2019)
Wheat (*Triticum aestivum*)	Panicle length and grain weight	ABE	*TaDEP1* and *TaGW2*	Li et al. (2020)
	Lipid metabolism	CBE	*TaLOX2*	Zong et al. (2017)
Maize (*Zea mays*)	Chromosomal segregation	CBE	*ZmCENH3*	Zong et al. (2017)

### Cytosine base editing system

A nuclease-deficient CRISPR system directs cytidine deaminase, which modifies the cytosine base. Deamination of cytosine produces uracil at the target location, which eventually transforms C-G into a T-A base pair without causing a double DNA strand break. The first-generation basic editor (BE1) was established in 2016 by Liu and others at Harvard University, United States. It is comprised of the cytosine deaminase APOBEC1 (from rat), which connects a linker with dCas9 through 16 unstructured XTEN amino acids. Base Editor (BE) and Target-AID (first cytosine base editing systems) employ rAPOBEC1 and PmCDA1, respectively, as deaminases and effectively insert alterations within the editing windows of 12–16 bases and 16–20 bases upstream of the PAM. The main limitation of BE1 is that uracil DNA glycosylase (UDG) often removes uracil, leading to a low editing efficacy. A series of upgraded basic editors have been designed, taking into account the limitations of BE1 and its low editing efficacy. When the C-terminal of the DNA component is combined with UGI (Uracil Glycosylase Inhibitor), a second-generation base editor (BE2) is formed: APOBEC-XTEN-dCas9-UGI (Komor et al., 2016). The activity of UDG is inhibited by the additive UGI, which catalyzes the deletion of UDNA from DNA inside the cells and commences the pathway of BER (base excision repair). The inhibition of BER generates a threefold increase in the efficacy of editing in human cells. Subsequently, a third-generation BE3 base editor was designed, which consists of an amalgamation of C-terminus and UGI *via* four amino acid linkers, and the fusion of the N-terminus of nickase Cas9 D10A with rAPOBEC1 *via* an XTEN linker (16 a.a) (Komor et al., 2016). Substitution of dCas9 with nCas9 (Cas9 nickase), cleaving the chain opposite decytidine is the main characteristic of the BE3 system. Therefore, the editing efficiency of BE3 is further improved sixfold compared with that of BE2. Compared with 0.1% in BE2, the application of nCas9 showed a 1.1% increase in indel frequency. The cytosine base editor conducts the automated conversion from C to T; however, the presence of multiple Cs in the catalytic window can cause off-target activity where C is converted into U. To minimize this error, multiple BE3 variants (using non-canonical PAM) were generated using different Cas9 variants. SpCas9 variants (such as VQR-BE3, VRER-BE3, EQR-BE3, and SaKKH-BE3) of *Staphylococcus aureus* target NGCG, NGAG, NGAN, and NNNRRT PAM sequences, respectively, and have improved the editing capability by 2.5 (Kim et al., 2017). In addition to SpCas9 variants, SaCas9 with an NNGRRT PAM sequence has also been applied in multiple research proposals, exhibiting higher efficacies. Various mutants of cytosine deaminase were produced, for example, YEE-BE3 and YEE-BE2, which increase the specificity of DNA and decrease off-target activity because of different editing window widths. YEE-BE3 showed the greatest editing efficiency within a narrow editing window width of (approximately 2 nt) (Kim et al., 2017). Activation-induced cytidine deaminase is another base editing method and Target-AID was developed and is composed of a cytidine deaminase pmCDA1 (from the southern eel) and a nickase (Cas9D10A) (Nishida et al., 2016). The Target-AID system, with increased efficiency, is used for targeted mutagenesis in human and mouse cells. Target-AID is a useful technique for generating numerous gene alterations in tomato and other crops in which a mutant population has been detected (Hunziker et al., 2020). The Target-AID technique may be used as an alternative whereby breeders can introduce allelic changes in many targets in a single line and generation. The editing effectiveness of BE3 and Target-AID are increased two- to threefold when UGI and nickase are used.

Further optimization of CBE was performed to reduce indel formation during base-editing, to improve editing efficiency, and to narrow the editing window. An improved fourth-generation base editing system (SaBE4 and BE4) was generated through the amalgamation of two UGI molecules with the N and C terminals of nCas9 *via* a 9-aa linker and with Cas9D10A and rAPOBEC1 *via* a 32-aa linker. The use of UGI prevents UNG from entering the uracil intermediate, inhibits the formation of BER, and limits unwanted products. Compared with SaBE4 and BE4, the average non-T product formation by SaBE4-Gam and BE4-Gam is reduced, and the C to T editing efficiency is improved. As a result, fourth-generation base editors may be used to successfully program from C to T, decreasing the creation of indels and improving product purity. Additionally, with the automated insertion of point mutations, deaminase is also used to build libraries of various point mutations located in target regions of genomes. To create local sequence diversity, two basic editing methods; TAM (Targeted AID-mediated mutagenesis) and CRISPR-X are utilized (Ma et al., 2016). Human AID is combined with dCas9 in the TAM system to obtain effective genetic diversity in animal cells. Excited AID variants are targeted by dCas9 in the CRISPR-X system to induce point mutations (local and diverse) (Hess et al., 2016). dCas9 was utilized as a DNA-targeting module and has been proved to be effective for gene editing. However, a major limitation is the requirement for G/C-rich PAM sequences. The first cytidine deaminase base editor (Cpf1-based) was developed by Li and others to improve the efficacy of base editing (Li et al., 2018). Cpf1 supports T-rich TTTN-PAM, produces 5 bp cohesive ends (Zetsche et al., 2015), and can analyze sgRNA, allowing it to be used in a variety of genome-targeting applications (Zetsche et al., 2017). A rat APOBEC1 domain is combined with UGI and catalytically inert dLbCpf1 (*Spirulina* bacterium Cpf1) to form dLbCpf1-BE0 (base editor). Before the PAM sequence, the base editor presents an editing window of 8–13 bps, with an efficiency of 20%–22%. As a result, Cpf1-based base editors can improve the base editing efficiency and provide various PAM sequence alternatives in the target gene (Mishra et al., 2020). Currently, the cytosine base editing technique is used for a variety of cereal plant traits (Table 3). The capability of the CBE3 method in rice was examined on three bases; firstly, (P2) in *OsPDS* that encodes phytoene desaturase; secondly, (S3); and thirdly, (S5) in OsSBEIIb that encrypts enzyme IIB restriction endonuclease (starch branching enzyme). Changes in a single nucleotide of the DNA were induced at P2, S3, and S5 target sites and the efficacies of inserted mutations were 1.0%, 10.5% and 19.2%, respectively. A high-amylose rice is generated during the destruction of the intron-exon boundary (Li et al., 2017; Lu and Zhu., 2017).

In rice, using the CBE3 system can produce stable SLR1 and NRT1.1B basic editing plants, with editing efficacies of 13.3% and 2.7%, respectively (Li et al., 2017). The use of NRT1.1B for effective editing in rice can increase the efficacy of nitrogen use (Hu et al., 2015). Through nCas9-cytidine deaminase fusion, the efficacy of targeted transformation from C to T in *ZmCENH3*, *TaLOX2*, *OsCDC48*, and *OsSPL14* genes as high as 43.5% in maize, wheat, and rice. rBE5 was created in rice through the linkage of Cas9n-NLS and hAID*D *via* a peptide linker, to edit *OSFLS2* and *Pi-d2* with efficacies of 57.0% and 30.8%, respectively. The *Pi-d2*-edited rice gene contains a point mutation that modulates the defense response to blast fungus, which is of significant agricultural importance (Ren et al., 2018). A3A-PBE (base editor) consists of UGI, human APOBEC3A, and nCas9. Unlike previous base editors, it can convert C to T in high GC content regions in maize, rice, and wheat within a window of 17 nt. (Zong et al., 2018). The CBE system converts C to T in rice with up to 80% frequency, using engineered SaCas9 and SpCas9 variants (Hua et al., 2019). These universal base modifying tools are expected to broaden the target range to include rice and other cereals.

### Adenine base editing system

Nicole Gaudelli, a researcher in David Liu’s laboratory, developed an adenine base editor that converts adenine to inosine (I), leading to an A to G conversion (Gaudelli et al., 2017). The first generation of ABE1.2 was produced using an XTEN linker (16a.a), with the fusion of a TadATadA* heterodimer and the N-terminus of nCas9. An NLS (nuclear localization signal) was combined with the C-terminus of nCas9. ABE, comprised of nCas9 and deoxy-adenosine deaminase. This connects with the target DNA sequence through guided RNA programming, unveiling small bubbles of ssDNA, within which a putative deoxyadenosine deaminase domain catalyzes the conversion of A to I, which is ultimately converted to a G**-**C base pair at the target site, after DNA replication (Wolf et al., 2002; Li et al., 2018). ABE has been further optimized to improve the editing efficiency by the fusion of a TadA (2.1)* domain to the C-terminus of nCas9 (D10A), the use of different TadA mutations, the use of an N-terminally inactivated TadA* subunit, or by changing the gap between nCas9 (D10A) and TadA (2.1)* subunit (linker length). The seventh generation of ABE (e.g., ABE7.10) was developed through protein engineering and extensive directed evolution, and it effectively converts the target A to G (approximately 50%) in human cells, with extremely high product purity (≥99.9%) and a very low incidence of indels (≤0.1%) (Koblan et al., 2018). In human cells, SpCas9-NG can also efficiently generate selective mutations at distinct NG PAM positions (Hua et al., 2019), providing an opportunity to expand the application of ABE editing. Currently, ABE-P1S (base editor) containing ecTadA* 7.10-nSpCas9 (D10A) shows an increased editing efficacy in rice, compared with the widely implemented fusion of 7.10-nSpCas9 (D10A) *ecTadA-ecTadA. The editing efficacy of other ABE systems (including SaCas9 or SaKKH-Cas9 variants) can also be enhanced using a fusion protein (ecTadA* 7.10-nCas9) (Hua et al., 2020). More effective ABE will promote its use in crop productivity to improve the grain size and yield of rice (Tiwari et al., 2020). The ABE base editor effectively regulates the alteration of A to G in cereal plants (Table 3) (Li et al., 2018). Together with the abovementioned CBE, ABE can induce four types of conversions (from A**-**T to G**-**C or C**-**G to T**-**A) at specific target sites in the genome, improving the base editing potential. rBE14 (base editor) of a TadA:TadA7.10 heterodimer guided by nCas9 (D10A) has been developed. In rice, it easily and efficiently converted A to T in *OsWRKY45*, *OsSERK2*, and *OsMPK6*, with corresponding rates of 62.3%, 32.1%, and 16.7% (Yan et al., 2018).

A novel ABE plant, based on the fusion of nCas9 and an improved tRNA adenosine deaminase, permitted the transformation of A to G with up to 59.1% frequency in wheat and rice and 7.5% in protoplasts. The amalgamation of nCas9 (D10A) and the recombinant ecTadA* 7.10 protein resulted in the development of the ABE-P1 plant. The impact of editing on ABE-P1 was estimated at the *OsSLR1* and *OsSPL14* gene loci of the rice, with editing efficacies of 12.5% and 26.0%, respectively. Four plant-compatible ABE binary vectors (pcABE) were developed through the fusion of nCas9 and various modified ecTadAs (Kang et al., 2018). A novel ABE adenosine base editor was designed to increase the number of targeted sites in the rice genome with the help of a SpCas9 variant. The target genes (*OsSPL17* and *OsSPL14*) presented editing efficacies of 45% and 25%, respectively. These findings indicate that ABE with SpCas9-NG plays an effective role in rice, expands the compatibility of PAM, and expands the application of ABE in crop plants (Hua et al., 2019).

### Glycosylase base editing system

**FIGURE 4:**
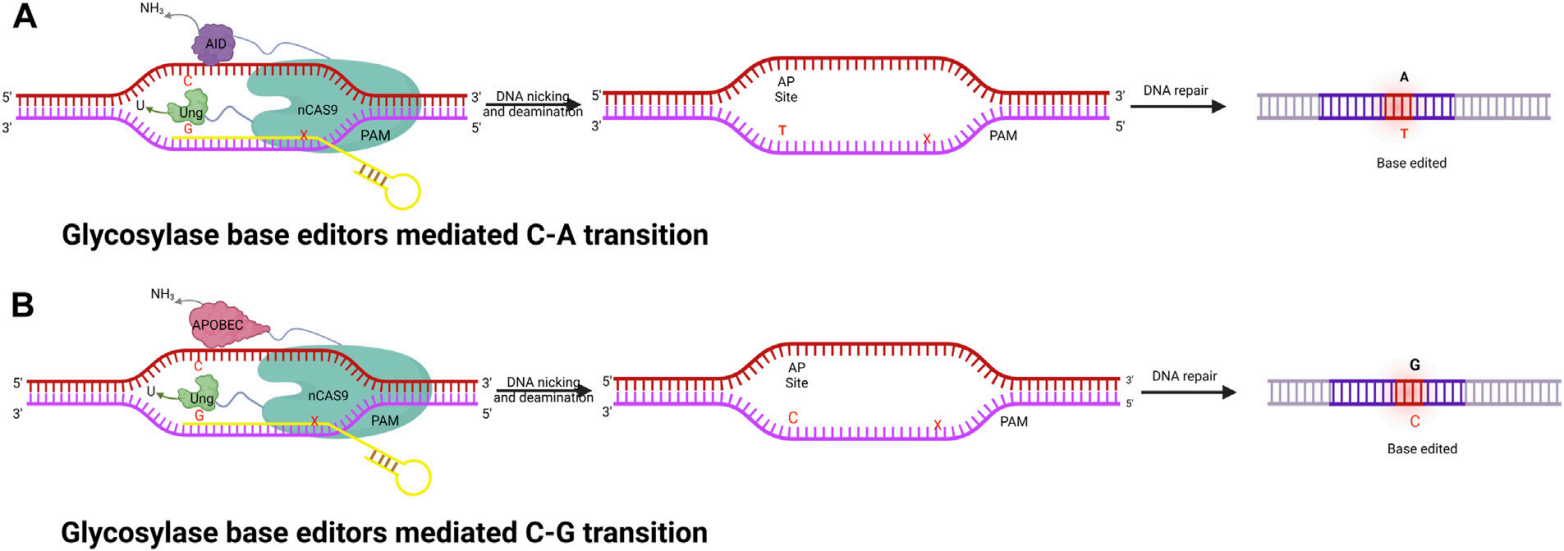
Illustration of fused nCas9, AID, and UNG enzyme complexes to perform a series of functions, including specific DNA binding, cleaving of the amine group from C, and creating AP sites followed by cellular repair to achieve specific base editing. **(A)** Glycosylase base editor–mediated C-A transition. **(B)** Glycosylase base editor–mediated C-G transition.

The base editors discussed thus far (CBE and ABE) can only catalyze base transitions (C-T and A-G). These BEs cannot generate base transversions; instead, they can only produce base transitions such as C-T (or G-A) and A-G (or T-C) swaps. To overcome these technology limitations, the Zhang and Changhao groups developed new base editors, namely, glycosylase base editors (GBE) (Zhao et al., 2021) (Figure 4). GBEs are made up of a uracil-DNA glycosylase (UNG), a Cas9 nickase, and a cytidine deaminases. UNG excises the U base produced by the deaminase, generating an apyrimidinic/apurinic (AP) site that begins the DNA repair procedure. As a new generation of base editing technology, GBE directly modifies the target base instead of relying on DNA replication. This technology further improves the base editing system, fills in any gaps in the different base editing systems, and realizes the arbitrary base editing of microbes for the first time. In wild-type *E. coli* strains, GBE editing technology has allowed the conversion of C-A with an accuracy of 93.8%. Any base editing (NBE) was also created, allowing any A, T, G, or C to be changed to any other base in a one, two, or three-step procedure. In addition to this, GBE allows the first C-G conversion in mammalian cells, with high position specificity and a narrow editing window. GBE achieved C-G conversions with a high specificity at the 6th C in an N20 sequence, which is different from other BE techniques (Zhao et al., 2021). A number of studies have been conducted to improve the performance of the C-G conversion (Liu et al., 2019; Koblan et al., 2021); however, in spite of this, the efficiency is still subpar and fluctuates greatly depending on the locus. Additionally, only a small number of GBEs with wider coverage were built. Thus, the continued development of GBE editors would facilitate various applications in genetic therapies and scientific research.

### C-G base editing system

Only one or two types of base substitutions may be accomplished using single base editors or dual deaminase-mediated base editors, respectively. Recently, a novel glycosylase base editor system (CGBE) system was developed, where Uracil-DNA glycosylase (UNG) is used instead of the uracil glycosylase inhibitor (UGI), to effectively initiate multiple base conversions, including C-A, C-T, and C-G. CGBE consists of a Cas9 nickase fused to a uracil DNA glycosylase (UNG) and cytidine deaminase. Architecturally, CBE and CGBE are comparable, the difference being that UNG is used in place of UGI. In addition, UNG excises the U base produced by deaminase, generating an AP site that begins the DNA repair procedure, which introduces indel mutations *via* an error-prone repair and replication mechanism, resulting in preferred insertion of G at the AP site and hence leading to C-G editing. The indels and the C-A and C-T conversions produced by CGBE are regarded as undesirable by-products for accurate base editing (Kurt et al., 2021; Zhao et al., 2021). On the contrary, it is also believed that these two by-products are advantageous when CGBE is employed to produce a saturated mutagenic population in a gene, because they broaden the range of BE outcomes. However, the reasons why G is selected over the other two bases are still a mystery.

Zhao et al. linked the amino N-terminus of nCas9 to APOBEC1 cytidine deaminases as well as UNG to the carboxy C-terminus (APOBEC1-nCas9-UNG) (Zhao et al., 2021), while Kurt et al. coupled both APOBEC1 and UNG at the N-terminus (UNG-APOBEC1-nCas9) (Kurt et al., 2021) to achieve C-G editing in mammalian cells. Kurt et al. used a mutant variant of rAPOBEC1 (R33A) for linkage. MiniCGBE was also developed by removing UNG from the original CGBE, with a comparable but slightly lower efficiency. In 2022, the Liang group combined an ABE and CGBE to create an AGBE system; a new type of dual deaminase-mediated base editing system that could concurrently achieve four different base conversions (A-G, C-T, C-A, and C-G) in addition to indels with a single sgRNA. High-throughput screening may be utilized with AGBEs to create saturated mutants to evaluate the effects of various gene mutation patterns, including single-nucleotide variations (SNVs) and indels (Liang et al., 2022)**.**

### RNA base editors (ADAR)

Zhang and his group were the first to develop RNA base editing to perform conversion of bases at the RNA level by using a catalytically inactive Cas13 (dCas13) and a naturally occurring ADAR (adenosine deaminase acting on RNA) to direct adenosine to inosine conversion (Cox et al., 2017; Yarra and Sahoo, 2021). The RESCUE and REPAIR systems for RNA editing have been introduced for mammalian cells; however, in plants no REPAIR and RESCUE mechanisms for RNA editing have been employed (Bharat et al., 2019). These new technologies will greatly boost the application of the CRISPR system in plant RNA editing. The application of these two systems to crop enhancement requires future exploitation in rice and other crops (Bharat et al., 2019).

### Targeting limitations of base editing

The target base must be present within a small base editing window for efficient base editing, and a specific PAM sequence is necessary (Gaudelli et al., 2017). This particular requirement for PAM is a strict restriction that reduces the editing efficiency in plant genomes. Modern ABE and CBE base editors are created with Cas9 variations, that can recognize PAM and NGG themes and because of this, the compatibility of PAM and the scope of basic editing has been increased (Endo et al., 2018; Nishimasu et al., 2018; Wang et al., 2019). The effectiveness of base editing is increased with these base editors, enhancing its applicability to a wide range of crops.

### Size of catalytic window

Cytosine deaminase (base editor) may edit any C base pair over a wide range of nucleotides (5–9 nt) and this becomes a major concern, resulting in a low specificity and editing efficacy. Therefore, it is necessary to develop a highly precise base editor with a small window size that can efficiently edit a single C in a certain catalytic window. These probes are created by removing non-essential nucleotides from deaminase and evaluating different lengths of proline-rich linkers in order to narrow the catalytic range and improve the efficiency. Furthermore, GBE editing technology has allowed conversion with very high accuracy; however, only a small number of GBEs with wider target ranges were built. Therefore, these high-efficiency and high-precision basic editors are effective tools in crop breeding.

### Off-target editing

Base editing (using CRISPR) is a recognized tool for base conversion. Previous research showed that the cleavage of on-target and off-target sites can be affected by different gRNA structures. Crystallography and single-molecule DNA curtain experiments showed that while the PAM site is essential to begin Cas9 binding, the sequence that corresponds to the 3′ end of the crRNA complementary recognition sequence, which is next to the PAM site, is also critical for subsequent Cas9 binding, activation of nuclease activities in Cas9, and R-loop formation. Off-target editing appears in this system when additional cytosines near the target base are edited. The activity of off-targets in BE3 is considerably decreased through the installment of mutations and the production of a high-fidelity base editor (HF-BE3) (Rees et al., 2017). CBEs, BE3, and HF1-BE3 have recently been found to cause unique and uncertain off-target alterations in rice (Jin et al., 2019). Such sudden alterations are commonly single nucleotide variants (SNV), from type C to type T. In order to mitigate these mutations, the literature suggests that it is obligatory to optimize UGI components and the cytidine deaminase domain. Furthermore, the modified CBE variant YEE-BE3 can be utilized in plants to decrease off-target editing (Jin et al., 2019).

### Prime editing

A precise gene editing technique, prime editing (PE), is capable of carrying out targeted, small insertions, deletions, and base swapping. This seems quite similar to current CRISPR techniques. PE results have previously been attained in a variety of ways. The capability to remove base pairs is a hallmark of knock-outs, while the ability to add specific base pairs in a precise manner is the premise underlying knock-ins. The ability to perform focus editing without causing double-stranded DNA breaks is what distinguishes PE from standard CRISPR (Chen et al., 2019). Precise and dependable editing technologies are required to create non-DSB and template-free, genome-edited organisms. PE and BE can respond to the demand for precise and effective non-DSB and template-free editing systems. However, base editors cannot generate transversions, insertions, or removals. Precise insertions may be accomplished without donor DNA templates. The restricted range of the present base editing conversions (C>T, G>A, T>C, and A>G) is expanded with prime editing to include all 12 combination swaps. PE is a complete solution, with little CRISPR procedural enhancements that significantly influence the outcome—a typical case of the whole being more than the sum of its parts.

**FIGURE 5:**
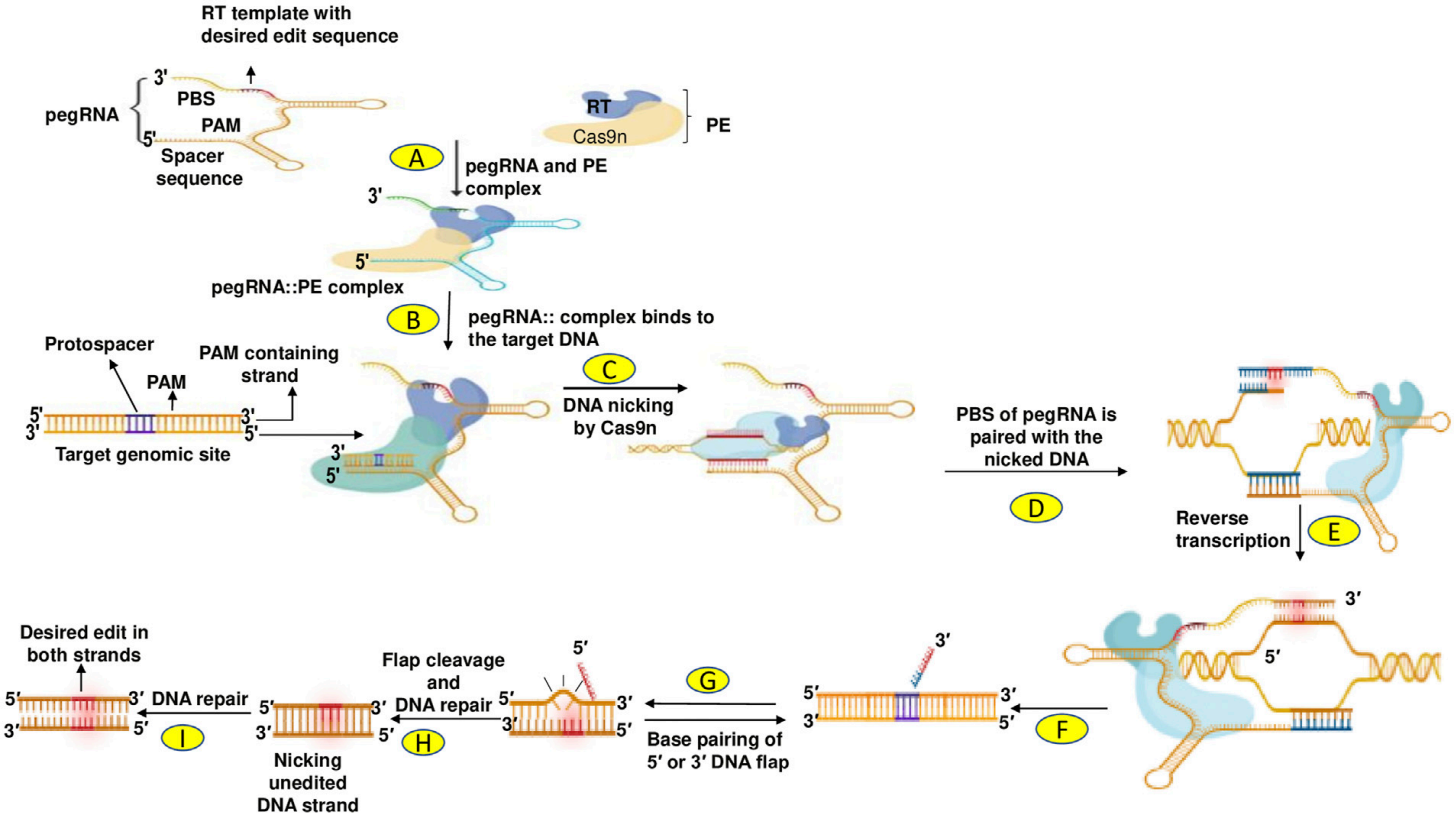
**(A)** Prime editors (PEs) are precise genome editing tools that directly write new genetic information into a specified DNA target site using a Cas9 nickase (nCas9; H840A) fused to an engineered reverse transcriptase (RT). **(B)** The RT is programmed with a prime editing gRNA (pegRNA) that specifies the target site and encodes the desired edit. PegRNA is a modified sgRNA with 3′ extension of the RT template and primer-binding site (PBS) sequences. **(C)** The nCas9 (catalytically impaired Cas9 harbouring a H840A mutation) is used to nick the editing strand of the double-stranded DNA target. **(D)** and **(E)** Next, the nicked strand is used for priming the reverse transcription of an edit-encoding extension (RT template) on the pegRNA directly into the target site. **(F)** This results in a branched intermediate consisting of two competing single-stranded DNA flaps. **(G)** The 3′ flap contains the edited sequence, whereas the 5′ flap contains the unedited sequence. **(H)** The 5′ flap is preferentially cleaved by structure-specific endonucleases such as FEN1 (Flap endonuclease 1: a central component of DNA metabolism) or 5′ exonucleases such as Exo1 (Human exonuclease 1) in mammalian cells. Ligation of the 3′ flap incorporates the edited DNA strand into the heteroduplex DNA containing one edited strand and one unedited strand. **(I)** Finally, to resolve the heteroduplex, DNA repair machinery permanently installs the desired edit by copying the information from the edited strand to the complementary strand.

PE developed by Anzalone et al. (2019) allows all types of mutations, including insertions and deletions, to be implemented in base-to-base conversions (Marzec et al., 2020). The prime editing technique has been improved and successfully used in mammalian cells and plants, allowing targeted indels (insertions and deletions) and point mutations without breaking double strands or DNA donor repair templates (Lin P. et al., 2020; Tang et al., 2020). PE is a dynamic and precise GE technique that uses a Cas9 endonuclease with catalytic impairments complexed to a designed transcriptase, configured with prime edit RNA (pegRNA). This governs the target site and induces the desired edits to create new genetic modifications directly at a specific DNA site. PE proofreading showed maximum or comparable effectiveness with fewer by-products and fixes targets by homology which is complementary to the basic editing strengths and weaknesses and induces much lower off-target mutations compared to Cas9. PE significantly increases the range and capability of GE, and can fix up to 89% of the recognized gene mutations in humans (Anzalone et al., 2019). Because PE provides a wide range of different genome modification types, it has strong potential for a variety of purposes including yield improvement, quality enhancement of products, and resistance to various abiotic and biotic stresses (Hassan et al., 2021). The principal events in prime editing are highlighted in Figure 5.

Lin Q. et al. (2020) adapted prime editors for use in plants through codon, promoter, and editing-condition optimization. The resultant suite of plant prime editors enable point mutations, insertions, and deletions in rice and wheat protoplasts. Regenerated prime-edited rice plants were obtained at frequencies of up to 21.8%. Two parts in this system are pivotal: The prime editor and a PE guide RNA (pegRNA). The pegRNA contains a site that includes a complementary DNA strand, a PBS (Primer Binding Site) (8–16 nt) sequence, and an RT-Template that carries the desired editing sequence, which may be replicated at the target location in the genome *via* reverse transcriptase. The prime editor, nickase Cas9 (Cas9n), possesses a mutant Cas9 protein that can cleave only one DNA strand. The editors also possess the necessary editing RT enzyme. The editor and pegRNA recombine during expression (transient or stable) and then travel toward the target site, led by pegRNA. The Cas9 nickase cleaves the PAM-containing strand to generate a single-strand DNA (ssDNA) flap; this process is directed by the target-specific pegRNA. The PBS, which itself is homologous to the ssDNA flap, intermixes with the RT blueprint and commences reverse transcription (RT), thus inserting sequences with the desired edit. After RT-mediated integration of the intended edit in the cleaved DNA molecule, the editing region comprises two duplicated ss-DNA flaps: an edited 3′ and un-edited 5′ DNA flaps. These ss-DNA flaps are eventually processed and integrated into the genome *via* endogenous DNA repair of the cell. The edited strand modifies the cleaved DNA strand by transferring sequence data from pegRNA, resulting in the development of a hetero-duplex with one unedited and one edited strand. The second cleave is produced by using a standard guide, RNA, in the unmodified DNA strand, which is then fixed by transferring base pair information from the edited stand, resulting in the desired edit being integrated into both strands of the DNA. PE systems have the capacity to edit the genome efficiently and accurately, hence playing an important role in GE.

**FIGURE 6:**
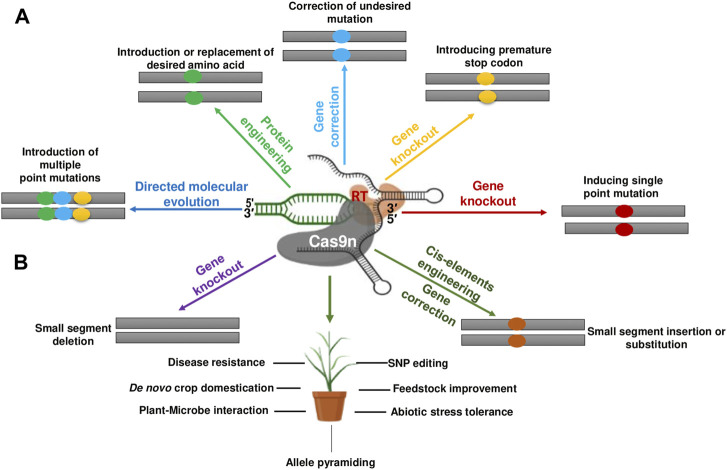
Prime editing-mediated genetic modifications and their potential use in cereals. **(A)** Various kinds genetic/sequence manipulations/modifications that are potentially possible through prime editing in plants. **(B)** Different applications of prime editing in various cereal crops. The rectangles specify mutation and different colors within them indicate different types of mutations. The yellow colored ovals denote the DNA segment inserted or replaced using prime editing. Cas9n, Cas9 nickase; SNP, single-nucleotide polymorphism; RT, reverse transcriptase.

A wide range of changes at genomic sites can be efficiently produced in plants (rice and wheat). However, for effective and accurate edits it is important to optimize pegRNA designs and editing conditions. Using plant prime editors (PPEs) is an alternative way to induce mutations that cannot be generated by other plant GE tools. In plants PE is less effective at inducing transitional point mutations than base editors. PPEs can possibly produce insertions, deletions, replacements, and transversions. PE in plants is a versatile tool as it holds the potential to advance novel plant breeding and functional genomics research. Prime editing-mediated genetic modifications, and their potential use in cereals, are shown in Figure 6. The efficiency of PE in rice is demonstrated by developing herbicide resistance through targeting of the *OsALS* gene; furthermore, a PE2 editor was used to edit *OsIPA* and *OsTB1* (Butt et al., 2019). Prime editors are promising tools as they precisely edit endogenous genes and transgenic lines in rice; however, a low prime editor efficacy has been reported in some rice transgenic lines. Jiang et al. (2020) pioneered the editing of two non-allelic targets using PE in maize, and confirmed the hypothesis that enhanced pegRNA expression could improve the editing efficiency. In summation, PE will broaden the scope and improve the capabilities of precise genome editing in important crops in future. Plant prime editing system optimization will empower the modification of crop genomes in a well-defined, cost-effective, and efficient manner, while fixing other superior agronomic traits. It should be noted that indels still occur in prime editing but with a frequency less than 1% in most cases. However, when using PE3, the indel frequency is generally less than 10%.

## Conclusion

CRISPR has become one of the most flexible genetic engineering tools in recent decades and is used for a variety of genome editing applications. In comparison to traditional procedures and transgenic techniques, GE approaches are more cost-effective, faster, and accurate in attaining the desired crop improvements. This technology presents many other diverse advantages over traditional breeding techniques such as overcoming incompatibility barriers and efficiently modifying the genome. In recent years, the CRISPR/Cas9 genome editing technique has become widely used in crop research, especially to develop resilient cereal crops such as rice, barley, wheat, and maize. Genomic sequencing has been utilized to apply CRISPR/Cas systems to modify genomes for producing abiotic and biotic stress tolerant crops and to enhance crop yields as desired. Although off-target impacts must be considered, altering agriculturally important cereal crops may lead to a promising “ever green revolution” in the near future, addressing concerns such as nutrition uptake, nitrogen fixation, photosynthesis, climate change, and biofuel production. Precise gene insertion and sequence substitutions still remain a major obstacle for molecular breeding using CRISPR/Cas systems. These hindrances have been overcome using BE and PE to precisely and effectively introduce non-DSB and template-free publishing systems. With the progress of new BE technologies and the further enhancement of precision, unparalleled prospects are available for both plant agricultural advancement and biological research.

## Future directions

Public acceptance and regulatory issues regarding CRISPR/Cas9 and its variants are still important issues to be resolved. The acceptance and wide application of this technology are still at the early stages and positive approaches to the related regulatory affairs may pave a way for global food and nutritional security. Looking ahead, at the global political level, people’s interest in food and nutrition security is increasing, significantly impacting cereal research. In 2015, a sustainable development target to eliminate hunger by 2030 was set by the UN. As a result of this increased worldwide interest, funding for cereal research is expanding, which helps to promote the development of numerous novel methodologies. Recently, a directed evolution platform, based on CRISPR/Cas has been designed for plants. For example, the SF3B1 spliceosome protein resists splicing inhibitors in rice; different degrees of resistance to inhibitors are conferred by such mutant versions. To increase production yields and to improve resistance to abiotic and biotic stresses, the directed evolution platform is useful for engineering crops. It provides the possibility of cultivating weather-resistant crops and can enhance global food security. Resistance genes can now be cloned more rapidly owing to genomic approaches, as evidenced by the exponential growth in the number of resistance genes cloned in different crops and the simultaneous publication of multiple resistance genes. With the currently available resources, technologies, and those under development, there may be a similar expansion in understanding the molecular mechanisms of various traits in grains. The vast evolutionary genetic engineering-based modifications offered by the CRISPR/Cas technology has enhanced the pace of crop improvement and has reduced the threat of food insecurity at the global level.

To increase the purview of base editing, previous studies designed SpCas9-NG, xCas9, and SpCas9s variants in plants to expand the number of sites recognized by Cas9 (Endo et al., 2019). Further expansion of three optimized editors (AncBE4max, BE4max, and ABEmax) was completed with the help of bpNLS (codon-optimized dimerization nuclear localization signal) and was implemented in rice (Wang et al., 2019). Compared with known CBE and ABE editors, these base editors showed higher editing efficiencies. These upgraded base editors are beneficial for the molecular breeding approach. In many crops, DNA base editing technology is implemented to correct point mutations that are related to several traits. Therefore, in future, it is necessary to adopt new engineering variants in order to strengthen the current base editors, improve the efficacy of editing, and broaden the purview of basic editing, so as to be used in a variety of crops.

Genome editing-based PE technology aims to reduce the negative effects linked to other genome editing methods such as CRISPR-Cas9 or BE. PE does not require HDR or DSB when using exogenous donor DNA templates. Presently, advancements have been made in increasing the effectiveness of genome editing using the PE ribonucleoprotein complex. PE systems have evolved across four generations, each achieving a greater level of effectiveness. Recent data imply that *in vitro* screening of pegRNAs is crucial before conducting *in vivo* research, because this supports the potential application of PEs in repairing a wide range of mutations. However, PE also presents several difficulties, such as undesired mutations brought on by the double cleaving technique required by PE3, limitations regarding large DNA insertions, and the choice of ideal PBS and RT template combinations. Therefore, substantial advancements are required for generating more efficient PPEs and extending their editing range. Because PE is still in its early developmental stages, much research has been focused on determining its efficiency and application in plant genome editing. The plant prime editing system can be used as an effective and universal technique in different crop species, providing a helpful tool for improving crops in a user-friendly manner. The modification of several precision genome editing tools for directed, accurate, and exact gene/allele replacement, in conjunction with classical breeding methods, will accelerate the breeding of diverse, superior crop varieties for maintainable agricultural development. Thus, we feel no hesitation in saying “To create a fully functional and high-precision genome editing tool, the prime editors must be optimized”.
